# Modeling of High-Efficiency Multi-Junction Polymer and Hybrid Solar Cells to Absorb Infrared Light

**DOI:** 10.3390/polym11020383

**Published:** 2019-02-22

**Authors:** Jobeda J. Khanam, Simon Y. Foo

**Affiliations:** Department of Electrical and Computer Engineering, FAMU-FSU College of Engineering, Tallahassee, FL 32310, USA; jk16c@my.fsu.edu

**Keywords:** short circuit current density (J_sc_), open circuit voltage (V_oc_), power conversion efficiency (PCE), fill factor (FF), organic/polymer solar cell (OSC), hybrid solar cell (HSC)

## Abstract

In this paper, we present our work on high-efficiency multi-junction polymer and hybrid solar cells. The transfer matrix method is used for optical modeling of an organic solar cell, which was inspired by the McGehee Group in Stanford University. The software simulation calculates the optimal thicknesses of the active layers to provide the best short circuit current (J_SC_) value. First, we show three designs of multi-junction polymer solar cells, which can absorb sunlight beyond the 1000 nm wavelengths. Then we present a novel high-efficiency hybrid (organic and inorganic) solar cell, which can absorb the sunlight with a wavelength beyond 2500 nm. Approximately 12% efficiency was obtained for the multi-junction polymer solar cell and 20% efficiency was obtained from every two-, three- and four-junction hybrid solar cell under 1 sun AM1.5 illumination.

## 1. Introduction

In the past few years, polymer solar cells (PSCs) have been attracting much attention due to their ease of processing, low cost, flexibility and lightweight nature compared to the traditional inorganic solar cells. The thickness of the materials used in polymer solar cells is limited due to their high absorption coefficient [1,2,3]. Although the organic solar cell (OSC) has a good future, its efficiency is still very low compared to the silicon solar cell [4]. There have been various methods implemented, such as annealing, device structure tuning and active material modification, to improve the efficiency of the PSC [5]. Among the various methods involving two or more organic junctions, the tandem structure is one of the most effective solutions. Furthermore, the photovoltaic devices using a mixture of inorganic nanoparticles and conjugated polymers, called hybrid solar cells, have gained popularity due to their ability to absorb near-infrared light. To optimize the device performance, it is essential to adjust the thickness of active layers used in tandem photovoltaic cells. The optimization of a tandem structure using trial and error experiments is costly and sometimes ineffective. Simulation is a more effective tool to create the best tandem device structure. The OSC device is mainly made of an organic layer sandwiched between two different metal electrodes. A bulk heterojunction (BHJ) organic solar cell consists of three components: An active layer, band alignment layer and electrodes. The active layer is a homogeneous mixture of donor and acceptor materials. The donor materials are generally conjugated polymers, whereas the acceptor materials are fullerene derivatives. The power conversion efficiency of the most promising structure, which is namely the P3HT:PCBM bulk heterojunction solar cell, has been reported to be 5% [6,7]. Benaissa et al. [8] showed that the hybrid solar cell absorbs light until 800 nm. The study by Islam [9] showed that the one-junction polymer solar cell with a P3HT:PCBM active layer can cover the 800 nm light spectrum with 2.9% efficiency. The study by Swapna et al. [10] showed that the one-junction polymer solar cell with MEHPPV:PCBM active layer covered the 800 nm light wavelength and only produced a current density of 6.82 mA/cm^2^. Wei et al. [11] showed the tandem (two-junction) PSC, with the PCPDTBT:PCBM and P3HT:PCBM active layers providing 9% efficiency. In most papers, the simulation and optimization were conducted for the one-junction PSC cells. In our paper, we showed that the multi-junction hybrid solar cell can absorb light beyond 2500 nm and cover the whole solar spectrum with 20% efficiency. We also created a tandem polymer with 12% efficiency. The device structure was arranged in such a way that the high band gap material on the top of the device and lower band gap materials on the bottom of the device were able to absorb the near-infrared spectrum of light. The tandem solar cell voltage was increased due to the multiple junctions and the current also increased as it covered the near-infrared spectrum, hence increasing efficiency. 

## 2. Materials and Methods

### 2.1. Theoretical Considerations

The organic and inorganic materials used in this simulation are shown in Table 1. To reduce the charge recombination, two different materials called the electron transport layer (ETL) and hole transport layer (HTL) are used, which collect the electron and hole, respectively, after charge separation in the interface [12]. 

### 2.2. Solar Cell Modeling

The optical transfer matrix theory describes the optical processes inside a thin film layer stack, which is used to evaluate the power conversion efficiency of the multi-junction photovoltaic cell [14]. The theory is explained by Roman et al. in detail [15]. When the light that has the energy of a photon with angular frequency ω strikes the organic solar cell, local energy dissipation takes place. The local energy dissipated in the organic solar cell at the point z is given by:

(1) $$Q\left(z\right)=\frac{1}{2}c\varepsilon_{0}\alpha n{\left|E\left(z\right)\right|}^{2}$$

where ε_0_ is the permittivity of the vacuum; n is the real index of refraction; α ($\alpha =\frac{4\pi k}{\lambda }$) is the absorption coefficient; k is the extinction coefficient; c is the speed of light; λ is the wavelength and E is the optical electric field at the point z.

G (z, λ), the exciton generation rate as a function of depth and wavelength, is given by:

(2) $$G\left(z,\lambda \right)=\frac{Q\left(z,\lambda \right)}{\hbar \omega }$$

where h is the plank constant and $\hbar =\frac{h}{2\pi }$.

Finally, one obtains the exciton generation rate at a depth of z by summing G (z, λ) over the visible spectrum:

(3) $$G\left(z\right)=\overset{\lambda =2500}{\underset{\lambda =300}{\sum }}G\left(z,\lambda \right)$$

For active layer thickness = t and assuming 100% internal quantum efficiency, the current density (mA/cm^2^) J_sc_ under AM1.5 illumination is given by:

(4) $$J_{\mathrm{sc}}=q\times \int_{0}^{t}G\left(z\right)\mathrm{dz}$$

We get the J–V characteristics from the following equation:

(5) $$J=J_{\mathrm{sc}}-J_{o}\;\left(\exp\left(\frac{q\left(V+J\cdot R_{s}\right)}{\mathrm{KT}}\right)-1\right)-\frac{V+J\cdot R_{s}}{R_{\mathrm{sh}}}$$

where $J_{o}=J_{\mathrm{sc}}/\left(\exp\left(\frac{q\cdot V_{\mathrm{oc}}}{\mathrm{KT}}\right)-1\right)$. J_o_ is the saturation current density (mA/cm^2^) under reverse bias; V_oc_ is the open circuit voltage; J_sc_ is the short circuit current density; q is the elementary charge; k is the Boltzmann constant; T is the temperature (K;) and V is the output voltage. We consider a serial resistance R_s_ = 0.1 Ω cm^2^, related to contact and bulk semiconductor resistances and a shunt resistance R_sh_ = 1000 Ω cm^2^. 

A very important parameter is the fill factor (FF), which is defined as:

(6) $$\mathrm{FF}={\;P}_{\max}/\left(V_{\mathrm{oc}}\cdot J_{\mathrm{sc}}\right)=\left(V_{\max}\cdot J_{\max}\right)/\left(V_{\mathrm{oc}}\cdot J_{\mathrm{sc}}\right)$$

The main parameter related to the cell performances is the power conversion efficiency (PCE) η:

(7) $$\eta =\frac{V_{\max}\cdot J_{\max}}{P_{\mathrm{in}}}=\mathrm{FF}\frac{V_{\mathrm{oc}}\cdot J_{\mathrm{sc}}}{P_{\mathrm{in}}}$$

where J_max_ and V_max_ are the current density and voltage that correspond to the maximum power P_max_ delivered by the solar cell. P_in_ is the incident photon flux (in mWcm^−2^) that corresponds to AM 1.5 (i.e., P_in_ is 100 mWcm^−2^).

The organic absorber materials are composed of a semiconductor-like materials where the band gap corresponds to the difference between the Lowest Unoccupied Molecular Orbital (LUMO) and the Highest Occupied Molecular Orbital (HOMO). The V_oc_ can be calculated by the following equation [16,17]:

(8) $$\mathrm{Voc}=\left[\frac{\mathrm{HOMO}\left(D\right)-\mathrm{LUMO}\left(A\right)}{q}\right]-0.3V$$

where HOMO (D) is the highest molecular orbital of donor material and LUMO (A) is the lowest molecular orbital of acceptor materials.

The HOMO and LUMO levels of the donor and acceptor used in the paper are shown in Table 2. The V_oc_ for the inorganic materials PbS is 0.6V [18]. The MATLAB program has been obtained from the McGehee Group MATLAB software. The transfer matrix method is used to calculate the transmission, reflection and attenuation by using the MATLAB software. The theory for the calculation is described in detail in the literature [19,20]. The input data are the names of materials which build the structure of the cell and their corresponding thickness (nm); as well as the real and imaginary parts of the complex refraction index of each material. From the Index_of_Refraction_library.xls file, the software will use the n and k refraction index for each selected material (See Supplementary Materials Figures S1–S8 for details) [21,22,23]. Under AM1.5 illumination (assuming 100% internal quantum efficiency) at all wavelengths, the software calculates generation rate (G) and J_sc_ by using Equation (1) to Equation (4) using these input data. By using the input n and k refraction index data for all the materials used in a cell for a wavelength range, we can determine how much light was absorbed and reflected by the cell. The J–V characteristics are calculated by using Equation (5). The fill factor and power conversion efficiency are calculated by Equation (6) and Equation (7), respectively. We can calculate the V_oc_ from Equation (8) by using the corresponding active layer HOMO and LUMO level. The AM 1.5 illumination used in the simulation [21], where we can see that the globe absorbs the solar irradiance (mW/cm^2^) up to a wavelength of 2500 nm.

## 3. Results

### 3.1. Results for Multi-Junction Polymer Solar Cells

Three types of multi-junction polymer solar cells were investigated. Based on different active layers, we stacked the cell. The J–V characteristics and variation of light intensity on the cell were analyzed. The power conversion efficiency was calculated from the J–V curve. These three types of multi-junction polymer solar cell were simulated.

#### 3.1.1. Type 1: Multi-Junction Polymer Solar Cell

**Figure 1:**
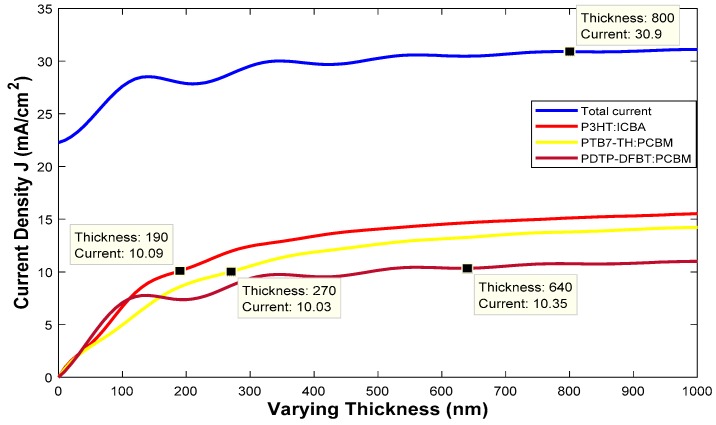
The thickness of the active layer is varied to obtain the optimal current for type 1 multi-junction polymer solar cells (PSC).

**Figure 2:**
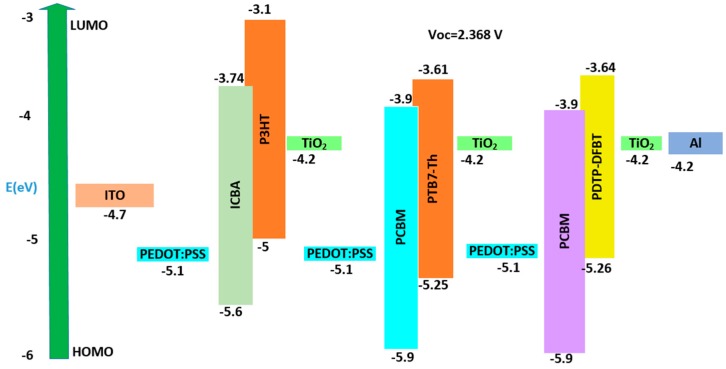
HOMO and LUMO band diagram for type 1 multi-junction PSC.

By using the high, medium and low bandgap organic materials, we arranged the stack for solar cells. The number of active layers determined the number of junctions. For the first multi-junction polymer solar cell, we used three active layers and hence it is called the three-junction polymer solar cell. During the first step, we determined the optical properties of a cell whose dimensions are given by: Glass/ITO (110 nm)/PEDOT:PSS (25 nm)/P3HT:ICBA (190 nm)/TiO_2_ (25 nm)/PEDOT:PSS (25 nm)/PTB7-Th:PCBM 270 nm)/TiO_2_ (25 nm)/PEDOT:PSS (25 nm)/PDTP-DFBT:PCBM (640 nm)/TiO_2_ (25 nm)/Al (200 nm). The thickness of the active layer affects the open circuit voltage (V_oc_) as well as the short circuit current (J_sc_) and thus the overall power conversion efficiency (PCE). To optimize various active layers, we use a general rule of thumb that decreasing the active layer thickness will increase the V_oc_ due to shorter diffusion length, while increasing the thickness will increase the J_sc_. Thus, we must optimize the cell to obtain the maximum performance. The active layer thickness was estimated by using the MATLAB code [21]. We can see in Figure 1 that the maximum total current that is possible from the type 1 solar cell is 30 mA/cm^2^. As the three junctions are connected in series, each junction should provide 10 mA/cm^2^ to obtain a maximum current of 30 mA/cm^2^ from the cell. We can see from Figure 1 that if we set certain thicknesses (190 nm for P3HT:ICBA active layer, 270 nm for PTB7-Th:PCBM active layer and 640 nm for PDTP-DFBT:PCBM active layer), we can obtain 10 mA/cm^2^ for each junction. Using the MATLAB code [21], we similarly calculated all the multi-junction polymer and hybrid cells optimized thickness. The HOMO and LUMO band diagram for the type 1 multi-junction PSC is shown in Figure 2. We can see from Figure 2 that with an active layer containing three different bandgaps, we determined that the open circuit voltage V_oc_ is 2.368 V.

**Figure 3:**
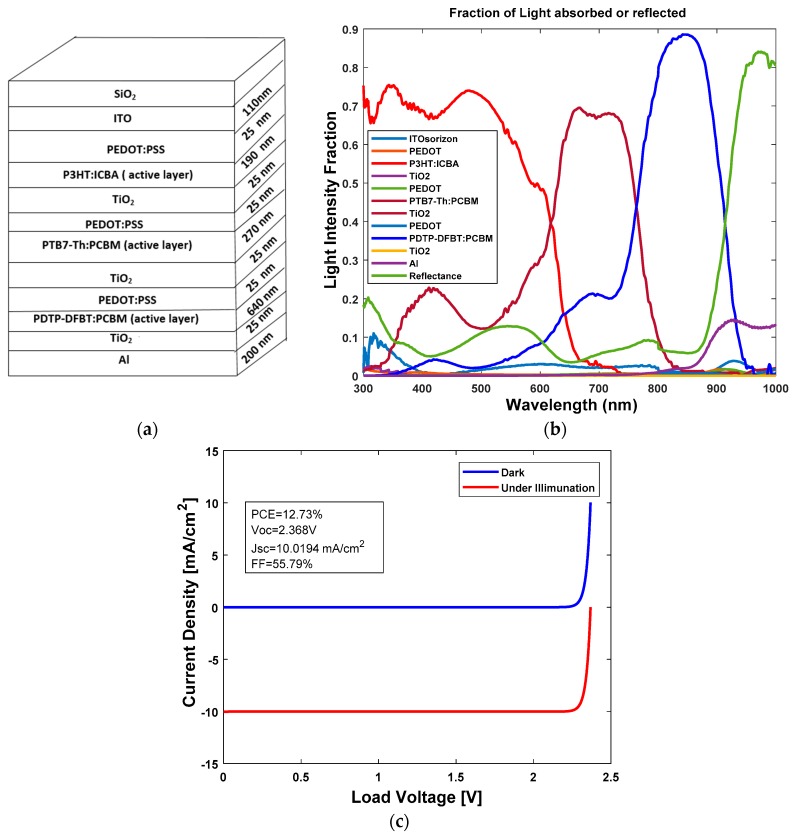
(**a**) Stack diagram; (**b**) variation of light intensity versus wavelength; and (**c**) J–V characteristics of three-junction organic solar cell (OSC) with front P3HT:ICBA, middle PTB7-Th:PCBM and rear PDTP-DFBT:PCBM active layers.

The stack diagram for the three-junction OSC is shown in Figure 3a. From Figure 3b, we can see that the three active layers of P3HT:ICBA (high bandgap), PTB7-Th:PCBM (medium bandgap) and PDTP-DFBT:PCBM (low bandgap) cover the solar spectrum with wavelengths of 300–1000 nm and the absorbed light intensity is above 70%. From the simulation, we determined that J_sc_ at each junction was 10.0194 mA/cm^2^. The J–V characteristics are shown in Figure 3c. A PCE of 12.73% was achieved with this configuration.

#### 3.1.2. Type 2: Multi-Junction Polymer Solar Cell

**Figure 4:**
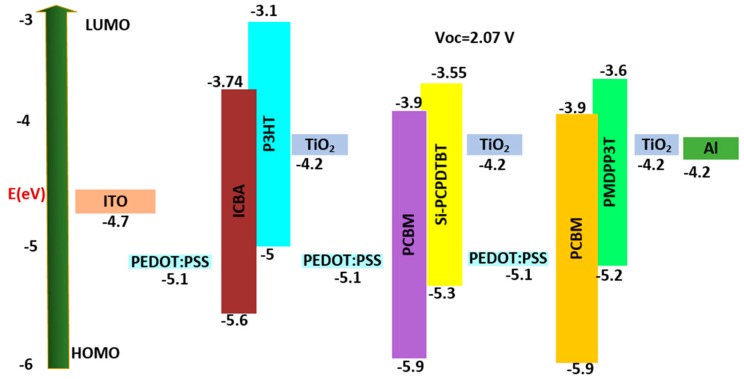
HOMO and LUMO band diagram for type 2 multi-junction PSC.

The optical properties of the second three-junction OSC were investigated. The dimensions of the cell are described as follows: Glass/ITO (110 nm)/PEDOT:PSS (25 nm)/P3HT:ICBA (235 nm)/TiO_2_ (25 nm)/PEDOT:PSS (25 nm)/Si-PCPDTBT:PCBM (290 nm)/TiO_2_ (25 nm)/PEDOT:PSS (25 nm)/PMDPP3T:PCBM (1000 nm)/TiO_2_ (25 nm)/Al (200 nm). The HOMO and LUMO band diagram for the type 2 multi-junction PSC is shown in Figure 4. We can see from Figure 4 that with an active layer of three different bandgaps, we determined that the open circuit voltage V_oc_ is 2.07 V.

**Figure 5:**
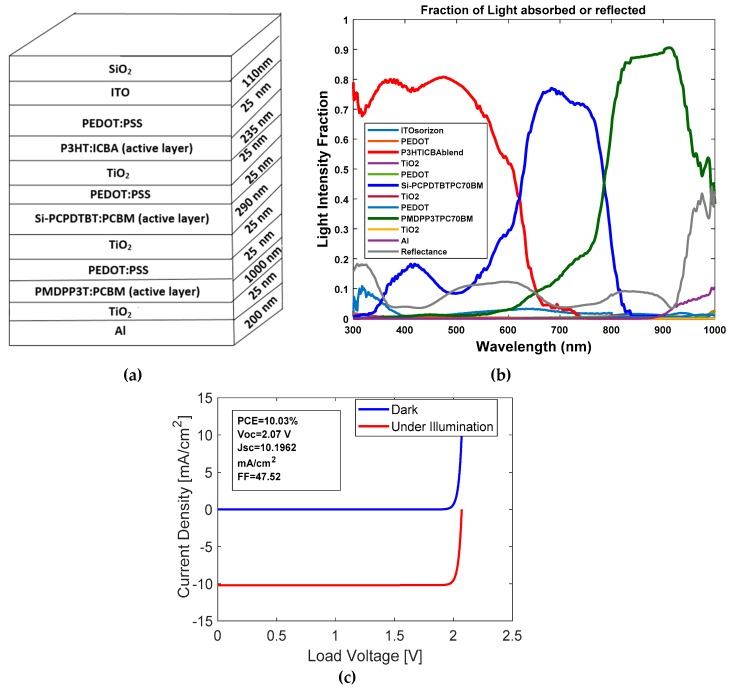
(**a**) Stack diagram; (**b**) variation of light intensity versus wavelength; and (**c**) J–V characteristics for OSC with P3HT:ICBA, Si-PCPDTBT:PCBM and PMDPP3T:PCBM active layers.

The stack diagram for the three-junction OSC is shown in Figure 5a. From Figure 5b, we can see that the three active layers of P3HT:ICBA, Si-PCPDTBT:PCBM and PMDPP3T:PCBM (low bandgap) cover the solar spectrum with wavelengths of 300–1000 nm and the light intensity is above 70%. J–V characteristics are shown in Figure 5c. From the simulation, we determined that J_sc_ at each junction was 10.1962 mA/cm^2^. A PCE of 10.03% was achieved with this configuration with FF of 47.52%.

#### 3.1.3. Type 3: Multi-Junction Polymer Solar Cell

**Figure 6:**
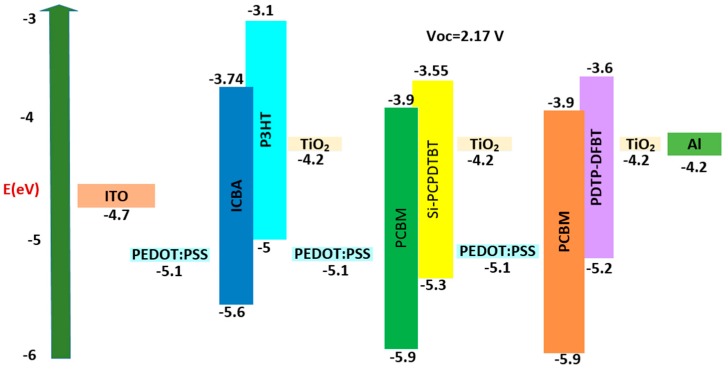
HOMO and LUMO band diagram for type 3 multi-junction PSC.

The third three-junction OSC was investigated. The dimensions of the cell were Glass/ITO (110 nm)/PEDOT:PSS (25 nm)/P3HT: ICBA (200 nm)/TiO_2_ (25 nm)/PEDOT:PSS (25 nm)/Si-PCPDTBT:PCBM (290 nm)/TiO_2_ (25 nm)/PEDOT:PSS (25 nm)/PDTP-DFBT:PCBM (1000 nm)/TiO_2_ (25 nm)/Al (200 nm). The HOMO and LUMO band diagram for the type 1 multi-junction PSC is shown in Figure 6. We can see from Figure 6 that with an active layer of three different bandgaps, we determined that the open circuit voltage V_oc_ is 2.17 V.

**Figure 7:**
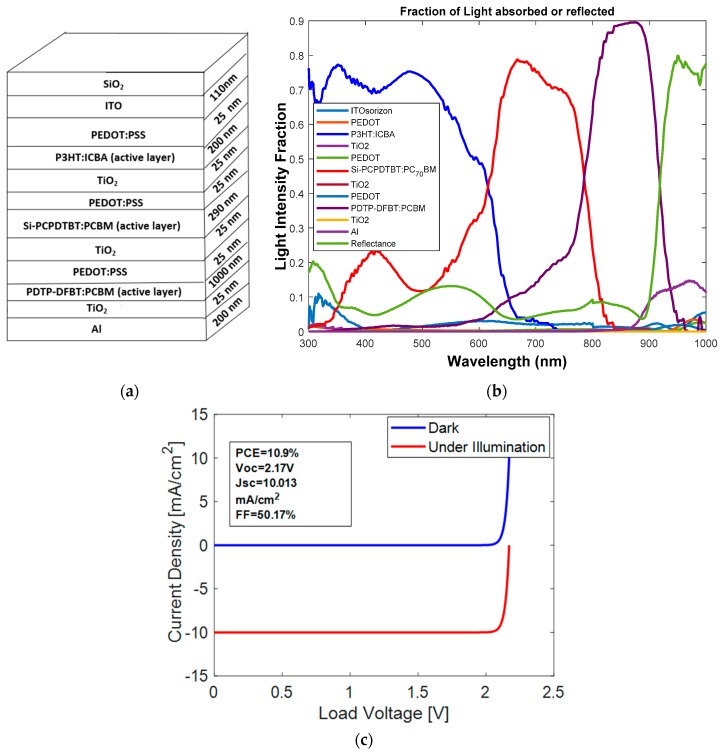
(**a**) Stack diagram; (**b**) variation of light intensity versus wavelength; and (**c**) J–V characteristics of OSC with P3HT:ICBA, Si-PCPDTBT:PCBM and PDTP-DFBT:PCBM active layers.

The stack diagram for the three-junction OSC is shown in Figure 7a. From Figure 7b, we can see that the three active layers of P3HT:ICBA, Si-PCPDTBT:PCBM and PDTP-DFBT:PCBM cover the solar spectrum with a wavelength in the range of 300–1000 nm and the light intensity is above 80%. J–V characteristics are shown in Figure 7c. From the simulation, we determined that the J_sc_ at each junction was 10.0130 mA/cm^2^. A PCE of 10.90% was achieved using this configuration with FF of 50.17%.

### 3.2. Results for Two-, Three- and Four-Junction Hybrid Solar Cell

Here, two-, three- and four-junction hybrid solar cells were investigated. Based on different active layers, we stacked the cell. The J–V characteristics and variation of light intensity on the cell were analyzed. The power conversion efficiency was calculated from the J–V curve. 

#### 3.2.1. Two-Junction Hybrid Solar Cell

**Figure 8:**
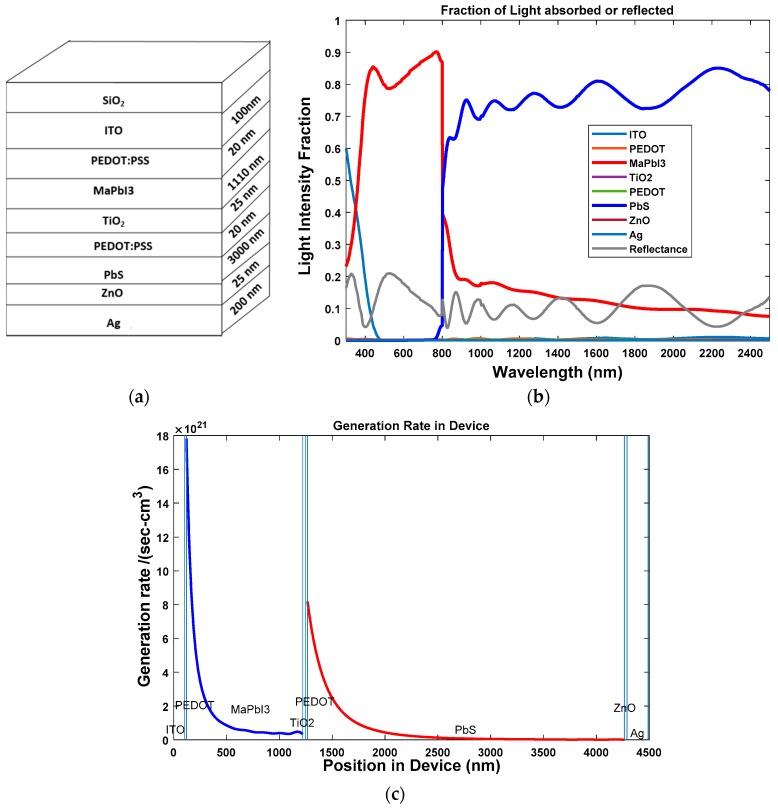
(**a**) Stack diagram; (**b**) variation of light intensity versus wavelength for HSC MaPbI_3_ and rear PbS active layers; and (**c**) exciton generation rate vs. position of device for two-junction hybrid solar cell.

First, the two-junction hybrid solar cell (HSC) was simulated. The lead sulfide (PbS) was used as a low bandgap material to absorb light beyond the infrared spectrum. The active layer MaPbI_3_ is organic and inorganic while PbS in HSC is inorganic in nature. During the first step, we determined the optical properties of a cell whose dimensions are given by: Glass/ITO (100 nm)/PEDOT:PSS (20 nm)/P3HT:ICBA (300 nm)/TiO_2_ (25 nm)/PEDOT:PSS (20 nm)/MaPbI_3_ (1110 nm)/TiO_2_ (25 nm)/PEDOT:PSS (20 nm)/PbS (3000 nm)/ZnO (25 nm)/Ag (200 nm). The stack diagram for the three-junction OSC is shown in Figure 8a. From Figure 8b, we can see that the two active layers of MaPbI_3_ and rear PbS active layers cover the solar spectrum with wavelengths of 300–2500 nm and the light intensity is above 80%. From Figure 8c, we can see that only the active layers are creating excitons.

#### 3.2.2. Three-Junction Hybrid Solar Cell

**Figure 9:**
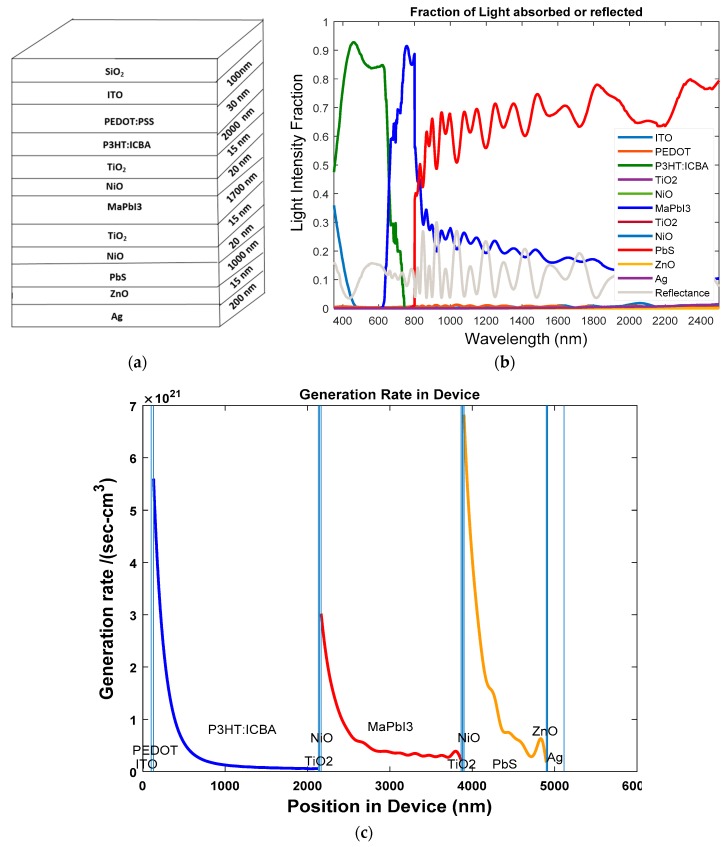
(**a**) Stack diagram; (**b**) variation of light intensity versus wavelength; and (**c**) exciton generation rate vs. position of device for three-junction hybrid solar cell.

The second solar cell investigated was the three-junction HSC. The active layer of P3HT: ICBA is organic but MaPbI_3_ is both inorganic and organic and PbS is inorganic in nature. During the first step, we determined the optical properties of a cell whose dimensions are given by: Glass/ITO (100 nm)/PEDOT:PSS (30 nm)/P3HT:ICBA (2000 nm)/TiO_2_(15 nm)/NiO (20 nm)/MAPbI_3_ (1700 nm)/TiO_2_(15 nm)/NiO (20 nm)/PbS (1000 nm)/ZnO (15 nm)/Ag (200 nm). The stack diagram for the three-junction OSC is shown in Figure 9a. From Figure 9b, we can see that the three active layers of P3HT: ICBA, MaPbI_3_ and rear PbS active layers cover the solar spectrum with wavelengths of 300– 2500 nm and the light intensity is above 80%. From Figure 9c, we can see that the three active layers are producing excitons.

#### 3.2.3. Four-Junction Hybrid Solar Cell

**Figure 10:**
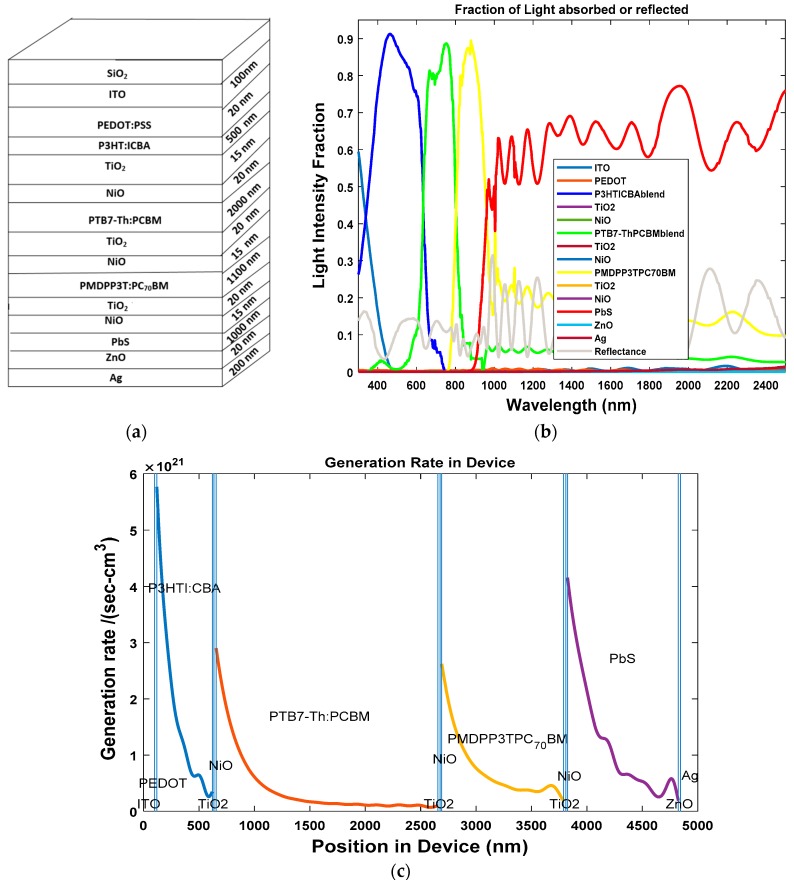
(**a**) Stack diagram; (**b**) variation of light intensity versus wavelength; and (**c**) exciton generation rate vs. position in device for two-junction hybrid solar cell.

The third solar cell that was investigated was the four-junction HSC. During the first step, we determined the optical properties of a cell whose dimensions are given by: Glass/ITO (100 nm)/PEDOT:PSS (20 nm)/P3HT:ICBA (500 nm)/TiO_2_ (15 nm)/NiO (20 nm)/PTB7-Th:PCBM (2000 nm)/TiO_2_ (20 nm)/NiO (15 nm)/PMDPP3T:PCBM (1100 nm)/TiO_2_ (20 nm)/NiO (15 nm)/PbS (1000 nm)/ZnO (20 nm)/Ag (200 nm). The stack diagram for the three-junction OSC is shown in Figure 10a. From Figure 10b, we can see that the three active layers of P3HT: ICBA, PTB7-Th: PCBM, PMDPP3T: PCBM and PbS cover the solar spectrum with wavelengths of 300–2500 nm and the light intensity is above 80%. From Figure 10c, we can see that the four active layers are producing excitons.

## 4. Discussions

### 4.1. Result Analysis for Three Types of Multi-Junction PSC and HSC

**Figure 11:**
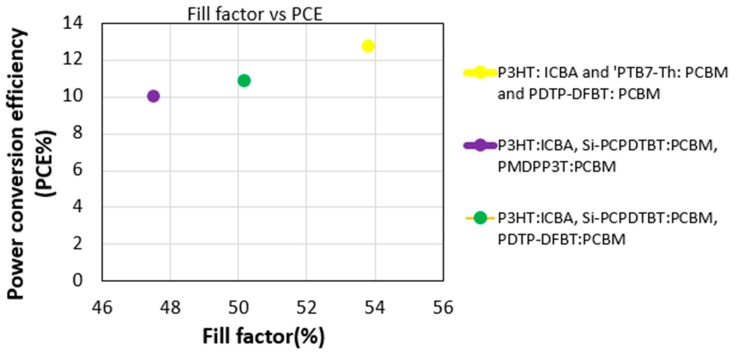
Efficiency vs. fill factor for multi-junction PSC.

**Figure 12:**
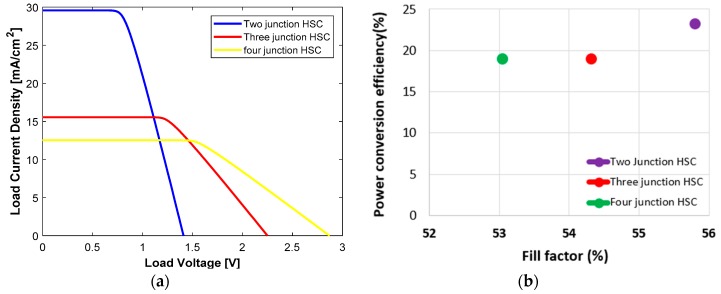
(**a**) J–V characteristics for two-, three- and four-junction hybrid solar cells. (**b**) Fill factor vs. efficiency for two-, three- and four-junction hybrid solar cells.

For the three types of multi-junction polymer solar cells, the efficiency compared to fill factor graphs are shown in Figure 11. In Figure 11, we observed that the efficiency for all three types of PSCs are above 10%. However, the P3HT:ICBA, PTB7-Th:PCBM and PDTP-DFBT:PCBM three-junction PSC has 12% efficiency. The J–V characteristics for two-, three- and four-junction hybrid solar cells are shown in Figure 12a, where we observed that two junctions produce a high J_sc_ of 30 mA/cm^2^ and four junctions produce a high V_oc_ of 2.8 V. In Figure 12b, we also observed that the power conversion efficiency is above 20% for the two-, three- and four-junction hybrid solar cells. In Figure 12b, we can see that MAPbI_3_ and PbS two-junction hybrid solar cell provides 22% efficiency with a 55% fill factor. As the fill factor was close to 50% for all cells, this proves that there was reasonable series resistance (Rs) and parallel resistance (R_sh_) during the calculation of current density.

### 4.2. Brief Fabrication Methodology for Multi-Junction Solar Cell

**Figure 13:**
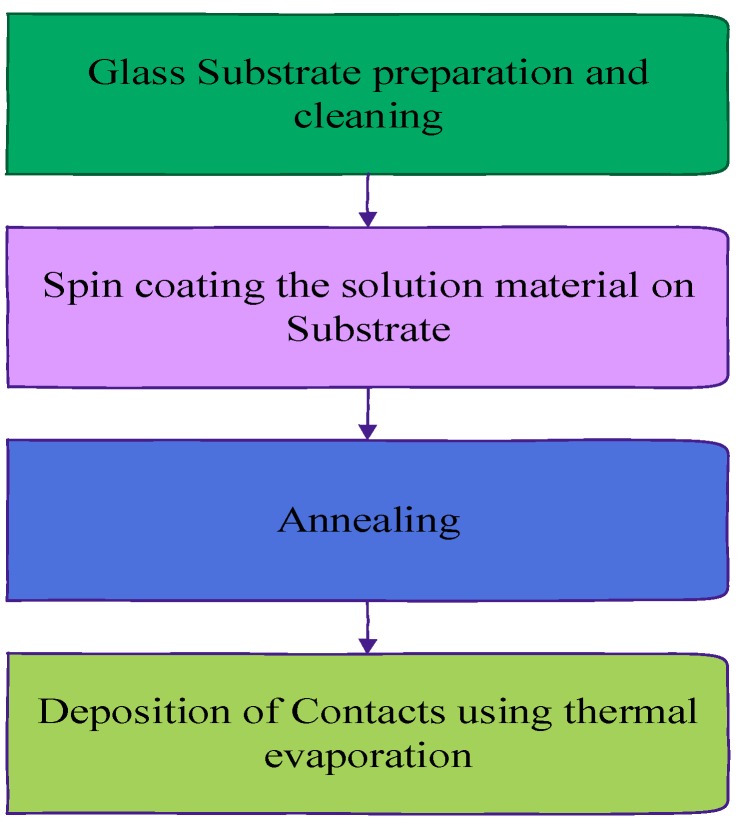
Fabrication methodology for multi-junction polymer and hybrid solar cell.

Future works should focus on the fabrication of the multi-junction PSC and HSC. The solution-processed multi-junction solar cells can be fabricated as shown in Figure 13. First, the ITO-coated glass electrode needs to be cleaned and prepared. After that, the spin coating is performed for each layer of multi-junction PSC and HSC. After each spin coating, annealing is performed. Finally, the cathode electrode is deposited using thermal evaporation.

### 4.3. Stability Analysis of PSC and HSC

Organic solar cells degrade extremely fast. The efficiency of organic solar cell changes according to moisture levels. Maxim et al. reported that water is the key factor determining the degradation in a solar cell. Thus, there should be encapsulation of the cell so that the oxygen and moisture cannot come in contact directly with the materials of the solar cell. Future studies should determine the variation in the efficiency of the fabricated PSC and HSC with humidity [24].

## 5. Conclusions

Theoretical settings have been shown to improve the efficiency of the organic solar cells, which were determined by optical modeling using the transfer matrix. We showed how we optimized the various active layers for a type 1 multi-junction PSC. In multi-junction cells, the junctions are connected in series, hence each junction current should be equal. By varying the active layer, we found the optimum current for each junction. The open circuit voltage was calculated by the HOMO and LUMO levels of the materials, which were used as active layers in OSCs and HSCs. The simulations were performed using the Stanford model. The performance of the device varied with the active layer thickness. From the results of our simulations, we showed that our multi-junction polymer solar cell and hybrid polymer solar cell can provide high efficiencies. The maximum PCE of 12.73% was achieved from the multi-junction polymer solar cell with the three active layers of P3HT: ICBA, Si-PCPDTBT:PCBM and PMDPP3T:PCBM. Lead sulfide (PbS) was shown to be the most promising low bandgap inorganic material, as it can absorb sunlight beyond the infrared spectrum. By using inorganic PbS and high band organic materials, we created a solar cell that absorbs sunlight beyond wavelengths of 2500 nm. The two-, three- and four-junction hybrid solar cells provided a PCE above 20%. The two-junction hybrid solar cell provided a high current of 30 mA/cm^2^ and the four-junction hybrid solar cell provided a high voltage of 2.8 V.

## 6. Patents

A patent has been filed with Florida State University and it is pending.

## Figures and Tables

**Table 1:** Description of organic and inorganic materials used in the simulation [13].

Symbol	Name; Description
SiO_2_	Silicon dioxide, glass
ITO	Indium tin oxide; electrode that collects hole/anode
PEDOT: PSS	Poly polystyrene sulfonate; HTL
P3HT	Poly(3-hexylthiophene-2,5-diyl), electron donor
ICBA	Indene-C60 bisadduct, electron acceptor
TiO_2_	Titanium (IV) oxide, ETL
PTB7-Th	Poly([2,6′-4,8-di(5-ethylhexylthienyl) benzo[1,2-b;3,3-b] dithiophene] {3-fluoro-2[(2-ethylhexyl) carbonyl] thieno[3,4-b] thiophenediyl}), electron donor
PCBM	[6,6]-phenyl-C71-butyric acid methyl ester, electron acceptor
PDTP-DFBT	Poly[2,7-(5,5-bis-(3,7-dimethyloctyl)-5H-dithieno[3,2-b:2′,3′-d] pyran)-alt-4,7-(5,6-difluoro-2,1,3-benzothia diazole); electron donor
Al	Aluminum; electrode that collects electron/cathode
PMDPP3T	Poly[[2,5-bis(2-hexyldecyl-2,3,5,6-tetrahydro-3,6-dioxopyrrolo[3,4-c] pyrrole-1,4-diyl]-alt- [3′,3″-dimethyl-2,2′:5′,2″-terthiophene]-5,5″-diyl]; electron donor
Si-PCPDTBT	Poly[2,1,3-benzothiadiazole-4,7-diyl[4,4-bis(2-ethylhexyl)-4H-silolo [3,2-b:4,5-b′] dithiophene-2,6-diyl]]; electron donor
MaPbI_3_	Methylammonium lead iodide; semiconducting organic–inorganic material
PbS	Lead (II) sulphide; semiconducting inorganic material
ZnO	Zinc oxide; ETL
Ag	Silver; electrode that collects electron/cathode
NiO	Nickel (II) oxide; HTL

**Table 2:** The HOMO and LUMO level of donor and acceptor of materials used in the simulation [13].

MATERIAL	LUMO (eV)	HOMO (eV)
PTB7-Th(donor)	−3.61	−5.25
PCBM (acceptor)	−3.9	−5.9
PMDPP3T(donor)	−3.6	−5.2
P3HT (donor)	−3.1	−5
PCPDTBT (donor)	−3.55	−5.3
MAPbI3	−3.93	−5.46
ICBA (acceptor)	−3.74	−5.6
Si-PCPDTBT (donor)	−3.55	−5.3
PDTP-DFBT (donor)	−3.64	−5.26

## Supplementary Materials

The following are available online at https://www.mdpi.com/2073-4360/11/2/383/s1, Figure S1: Refrative index (n,k) vs wavelngth for PbS, Figure S2: Refrative index (n,k) vs wavelngth for MAPbI_3_, Figure S3: Refrative index (n,k) vs wavelngth for P3HT:ICBA blend, Figure S4: Refractive index (n,k) vs wavelngth for PMDPP3T:PCBM blend, Figure S5: Refractive index (n,k) vs wavelength for PTB_7_: PCBM blend, Figure S6: Refrative index (n,k) vs wavelngth for PDTPDFBT: PCBM blend, Figure S7: Refractive index (n,k) vs wavelength for Si-PCPDTBT: PCBM blend, Figure S8: Refractive index (n,k) vs wavelength for PCDTBT: PCBM blend.
