# Supplementary material for: Modeling of High-Efficiency Multi-Junction Polymer and Hybrid Solar Cells to Absorb Infrared Light
Source: Polymers (Basel). 2019 Feb 22;11(2):383. doi: 10.3390/polym11020383 (PMC6419226; doi:10.3390/polym11020383)
Supplement: Supplementary file 1 [file polymers-11-00383-s001.pdf]

Refraction Index for all active layers

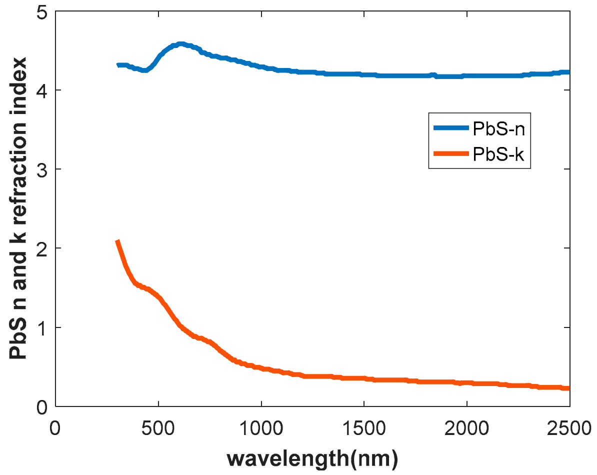

Fig S1: Refrative index (n,k) vs wavelngth for PbS

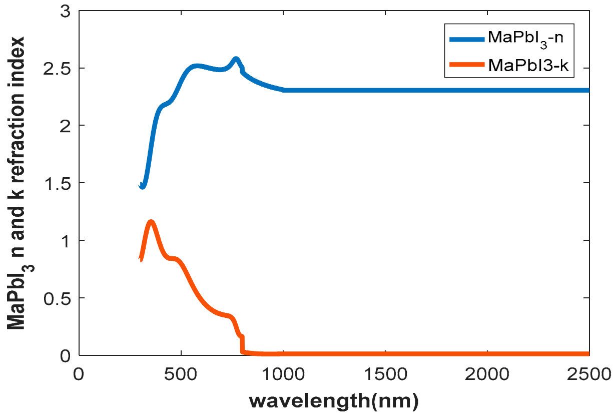

Fig S2: Refrative index (n,k) vs wavelngth for $MAPbI_3$

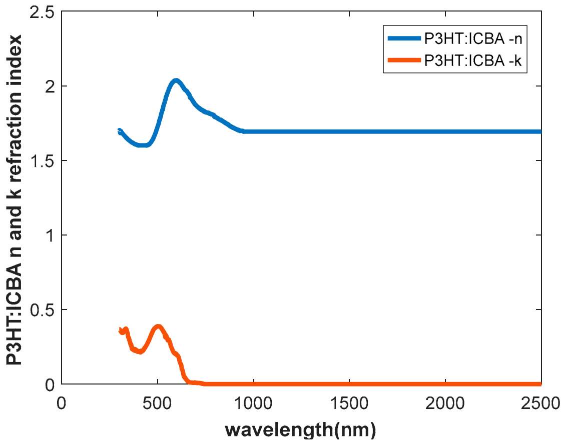

Fig S3: Refrative index (n,k) vs wavelngth for P3HT:ICBA blend

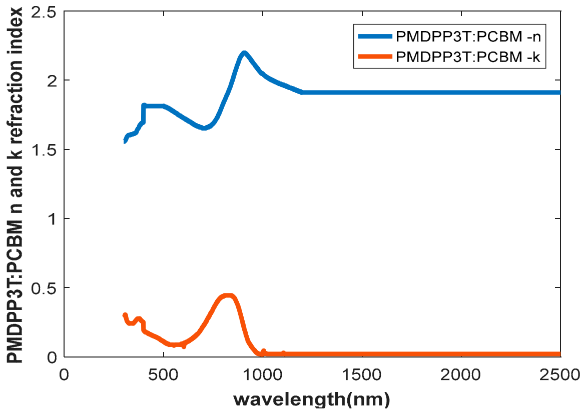

Fig S4: Refractive index (n,k) vs wavelngth for PMDPP3T:PCBM blend

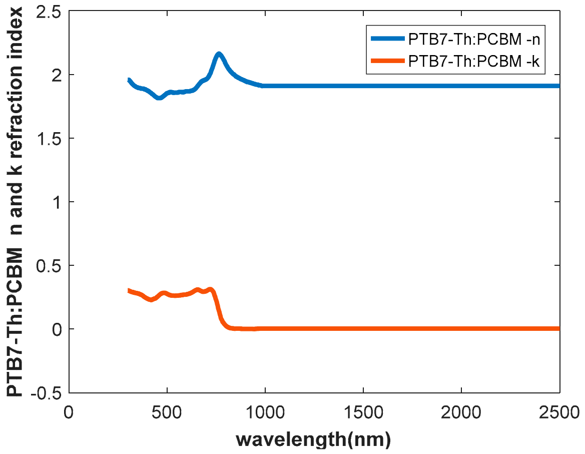

Fig S5: Refractive index (n,k) vs wavelength for PTB_7: PCBM blend

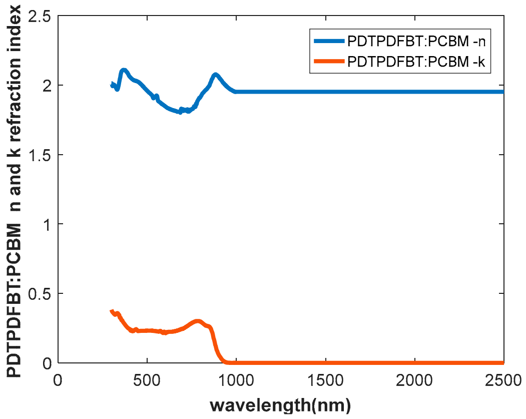

Fig S6: Refrative index (n,k) vs wavelngth for PDTPDFBT: PCBM blend

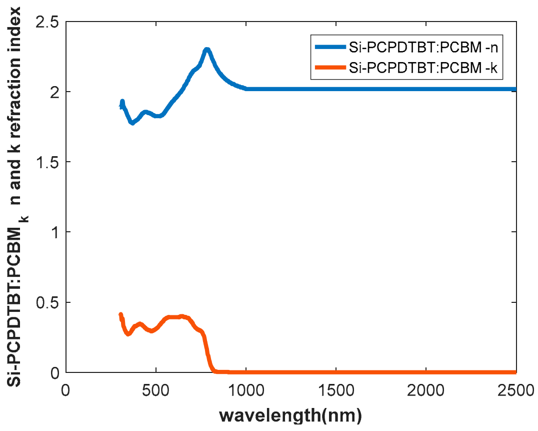

Fig S7: Refractive index (n,k) vs wavelength for Si-PCPDTBT: PCBM blend

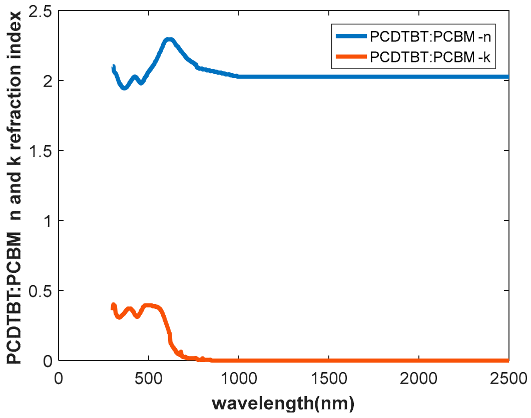

Fig S8: Refractive index (n,k) vs wavelength for PCDTBT: PCBM blend
